# Rapid determination of chemical composition and classification of bamboo fractions using visible–near infrared spectroscopy coupled with multivariate data analysis

**DOI:** 10.1186/s13068-016-0443-z

**Published:** 2016-02-09

**Authors:** Zhong Yang, Kang Li, Maomao Zhang, Donglin Xin, Junhua Zhang

**Affiliations:** Research Institute of Forestry New Technology, Chinese Academy of Forestry, 100091 Beijing, China; Research Institute of Wood Industry, Chinese Academy of Forestry, 100091 Beijing, China; College of Forestry, Northwest A&F University, 3 Taicheng Road, 712100 Yangling, China

**Keywords:** Bamboo, Botanical fractions, Quantitative analysis, Qualitative classification, Multivariate analysis

## Abstract

**Background:**

During conversion of bamboo into biofuels and chemicals, it is necessary to efficiently predict the chemical composition and digestibility of biomass. However, traditional methods for determination of lignocellulosic biomass composition are expensive and time consuming. In this work, a novel and fast method for quantitative and qualitative analysis of chemical composition and enzymatic digestibilities of juvenile bamboo and mature bamboo fractions (bamboo green, bamboo timber, bamboo yellow, bamboo node, and bamboo branch) using visible–near infrared spectra was evaluated.

**Results:**

The developed partial least squares models yielded coefficients of determination in calibration of 0.88, 0.94, and 0.96, for cellulose, xylan, and lignin of bamboo fractions in raw spectra, respectively. After visible–near infrared spectra being pretreated, the corresponding coefficients of determination in calibration yielded by the developed partial least squares models are 0.994, 0.990, and 0.996, respectively. The score plots of principal component analysis of mature bamboo, juvenile bamboo, and different fractions of mature bamboo were obviously distinguished in raw spectra. Based on partial least squares discriminant analysis, the classification accuracies of mature bamboo, juvenile bamboo, and different fractions of bamboo (bamboo green, bamboo timber, bamboo yellow, and bamboo branch) all reached 100 %. In addition, high accuracies of evaluation of the enzymatic digestibilities of bamboo fractions after pretreatment with aqueous ammonia were also observed.

**Conclusions:**

The results showed the potential of visible–near infrared spectroscopy in combination with multivariate analysis in efficiently analyzing the chemical composition and hydrolysabilities of lignocellulosic biomass, such as bamboo fractions.

**Electronic supplementary material:**

The online version of this article (doi:10.1186/s13068-016-0443-z) contains supplementary material, which is available to authorized users.

## Background

Bamboo is a major non-wood forest product and wood substitute, which is considered as one of the important resources in wood industry to replace woody resources. Bamboo represents a significant basic material, particularly in Asia, where it is used for construction, pulp and paper, food, combustion, and furniture [1]. About 300 different species of bamboo are known to grow in Asia [2]. What a pity, it is that the residues of bamboo after processing industry are wasted and not fully utilized. In recent years, bamboo has been researched for different kinds of applications, including its use as a biomass feedstock for production of biofuels and chemicals.

The properties of bamboo directly affect the use of bamboo, such as anatomical properties, of which fiber length affects the strength properties of paper [3]. Bamboo’s physical and mechanical properties are closely related to structural application [4]. Therefore, the study on the anatomical, physical, and mechanical properties is important for the selection of suitable bamboo for industrial use, construction, and housing [5]. The physical properties of the bamboo are significantly affected by the distribution and contents of cellulose, hemicellulose, and lignin. For example, the difference in lignin contents results in a significant difference in physical and mechanical properties between mature bamboo and juvenile bamboo [6]. In addition, juvenile bamboo belongs to immature bamboo shoot becoming inedible owing to the increase of rough fiber [7] and not being used as raw materials for furniture, construction, and pulp and paper due to the weakness in mechanical properties. On the other hand, the time for the growth of juvenile bamboo to mature bamboo is relatively short. After juvenile bamboo emerges in early April or thereabouts, it typically reaches a mature state in less than two months with an average height of 15 m and an aboveground carbon mass of 3.95 kg [8], which makes it difficult to distinguish between juvenile bamboo and mature bamboo. So it is significant to discriminate them quickly and accurately.

Recently, many attentions have been focused on the utilization of bamboo material for production of biofuels, such as bioethanol [9–11]. However, it was found that the structural properties significantly affect the efficiency of cellulose conversion. Bamboo fractions after pretreatment with aqueous ammonia (with low lignin content) showed better enzymatic digestibility than those after pretreatment with dilute acid (with high lignin content), indicating that bamboo with low content of lignin was more susceptible to cellulases [10]. Additionally, higher hydrolysis yields obtained from bamboo shoots than mature bamboo also confirmed the fact that cellulases are prone to hydrolysis of bamboo with low content of lignin. [11]. Therefore, it is feasible in theory to generally evaluate the enzymatic digestibility of lignocellulosic biomass based on the chemical composition of the material. As bioethanol and biomass power are gradually valued highly, a rapid composition analysis method that evaluates the hydrolysis yield of sugar is desired for bioethanol manufacturers and bio-power producers [12]. Traditional methods for chemical characterization of biomass feedstock, such as wet chemical analysis, are expensive (labor intensive) and time consuming. Additionally, the disposal of waste chemicals resulted from wet chemical analysis is also a concern. Quantitative spectroscopy provides a fast and reliable alternative for traditional analytical methods to determine the chemical composition of a sample. Near infrared spectroscopy (NIR) has the advantages of fast analysis, no damage to the sample, and good repeatability and accuracy [13].

Early, NIR spectroscopy was applied to agriculture and food factory [14, 15]. It has been generally used in the research of wood science. For example, NIR spectroscopy was used to investigate wood properties, such as chemical [16–18], physical [19, 20], and mechanical properties [21, 22]. In recent years, NIR spectroscopy has been applied in bamboo. Huang et al. [23] evaluated the klason lignin content of Moso bamboo based on the visible and near infrared spectroscopy. Xu et al. [24] rapidly determined bamboo shoot lignification associated with crude fiber content and firmness using Fourier transform near infrared spectroscopy. Lu et al. [25] determined flavonoids and phenolic acids in the extract of bamboo leaves using near infrared spectroscopy and multivariate calibration. Wu et al. [26] applied near infrared spectroscopy for the rapid determination of antioxidant activity of bamboo leaf extract. However, there are hardly any studies demonstrating quantitative prediction of main chemical composition of bamboo, enzymatic digestibility of the material, and qualitative classification of bamboo fractions based on NIR spectroscopy.

In this work, the bamboo samples have been manually separated into five bamboo fractions, namely bamboo green, bamboo timber, bamboo yellow, bamboo node, and bamboo branch. Their chemical composition was determined by conventional wet chemical analyses. Based on visible–near infrared spectra acquired on the biomass, partial least squares (PLS) regression was used for quantitatively analyzing the chemical composition of bamboo and determining the general hydrolysabilities of the materials, and partial least squares discriminant analysis (PLS-DA) was used for qualitatively discriminating between mature bamboo and juvenile bamboo, classifying separated bamboo fractions and sugar yield level.

## Results and discussion

### Quantitative prediction of chemical composition

**Fig. 1:**
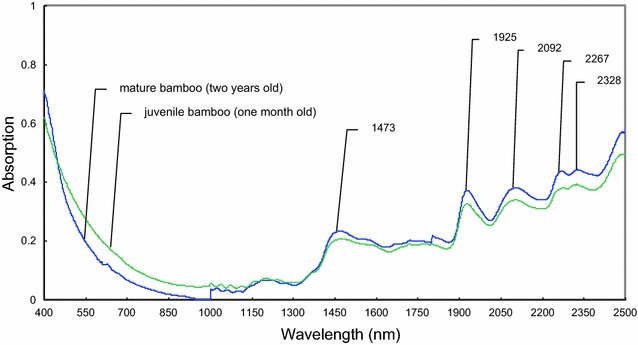
Visible–near infrared mean spectra from original data of bamboo timber fractions. Data of one spectrum were collected from 2-year-old mature bamboo and those of another spectrum were collected from one-month-old juvenile bamboo

The visible–near infrared mean spectra of bamboo timber fraction of one-month-old juvenile bamboo and 2-year-old mature bamboo are shown in Fig. 1. Many absorption band peaks occurred in the wavelength region of 1100–2500 nm, including prominent peaks at around 1473, 1925, 2092, 2267, and 2328 nm. The peak at 1473 nm was primarily attributed to the first overtone O–H stretching of cellulose. The strong peak at approximately 1925 nm was primarily attributed to the O–H asymmetric stretching and O–H deformation from water [27]. The O–H and C–H deformation and O–H stretching vibration of cellulose and xylan were indicated by spectral changes at 2092 nm. Further, the overtone of O–H stretching and C–O stretching from lignin at 2267 nm also showed a change in absorption [28]. The C–H deformation and C–H stretching vibration of xylan were indicated by spectral changes at 2328 nm.

**Fig. 2:**
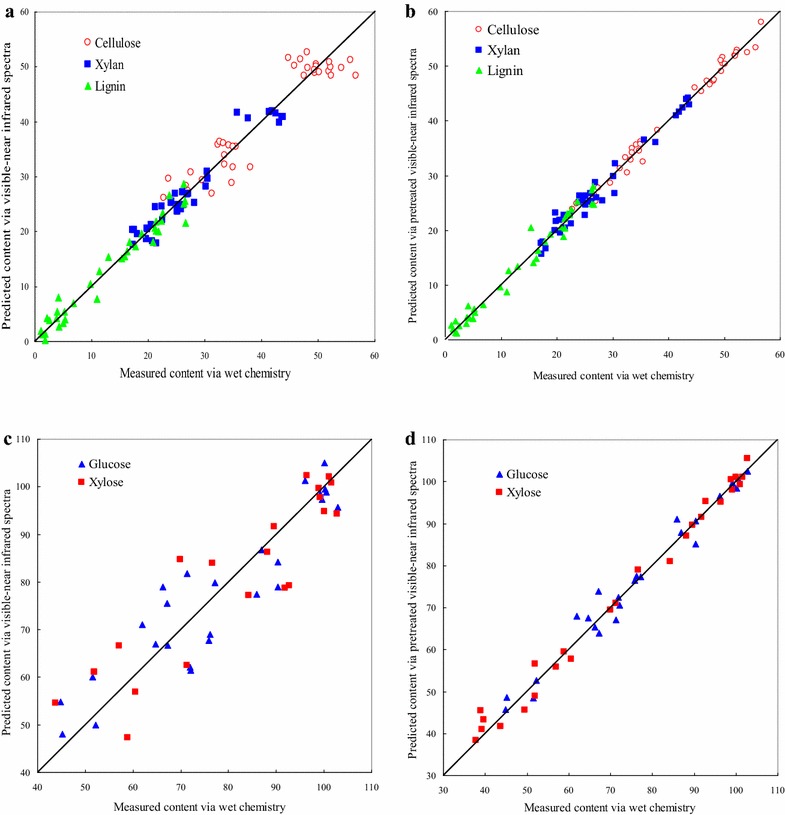
Chemical contents predicted by visible–near infrared spectra vs. those measured by wet chemistry: **a** non-pretreatment (cellulose, xylan, and lignin); **b** first derivative pretreatment (cellulose, xylan, and lignin); **c** non-pretreatment (glucose and xylose); and **d** first derivative pretreatment (glucose and xylose)

**Fig. 3:**
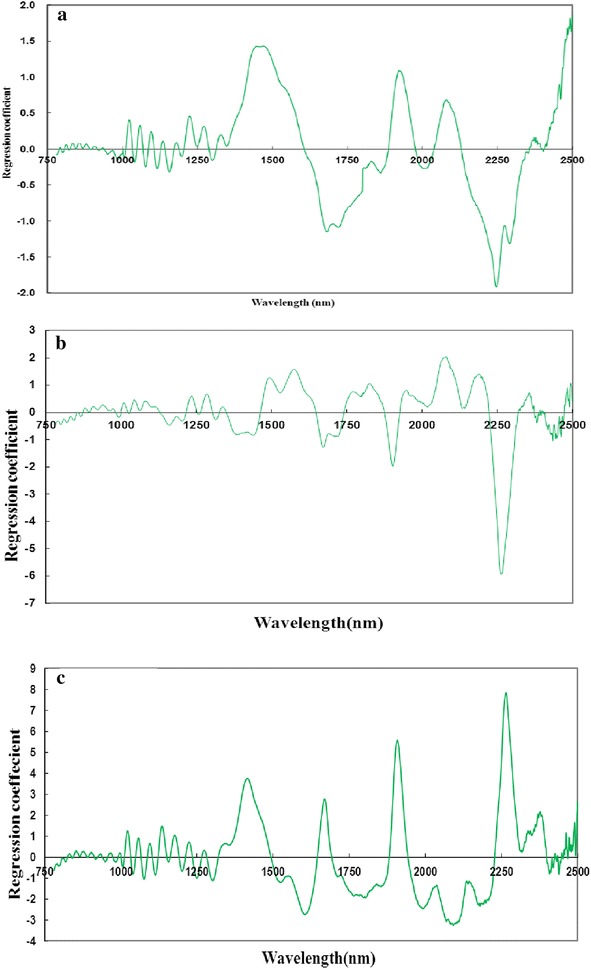
Regression coefficient plots of chemical composition of bamboo based on raw NIR spectra (780–2500 nm): **a** regression coefficient plot of cellulose; **b** regression coefficient plot of xylan; and **c** regression coefficient plot of lignin

Thirty six samples are prepared for quantitative analysis of the three chemical compositions. The predicted chemical contents (cellulose, xylan, and lignin) via raw visible–near infrared spectra vs. wet chemistry measurements as results generated by the PLS regression procedure are presented in Fig. 2a, together with a target line. The experimental values and estimated values for cellulose, xylan, and lignin are presented in Additional file 1: Table S1. Generally, correlation was high between predicted chemical contents via PLS regression and wet chemistry measurements, demonstrating the feasibility of PLS regression in predicting the chemical composition of bamboo fractions. Results of calibration and PLS1 and PLS2 prediction models for the quantitative compositional analysis of bamboo using raw spectra (visible light, NIR, and visible–near infrared spectra) are presented in Table 1 and Additional file 1: Table S2, respectively. As observed in Table 1, when raw spectral regions were in NIR range (780–2500 nm) and visible–near infrared range (400–2500 nm), the ratio of root mean square error of prediction (RMSEP) to standard deviation (SD) (RPD) values were almost more than 2.5 in PLS1 model, indicating that the PLS1 models provided a good prediction. At the same time, the values of the range error ratio (RER) in PLS1 models were more than 6.1. Besides, the results of the prediction models exhibited high coefficient of determination in calibration (R^2^c) values ranged between 0.88 and 0.96, low root mean square error of calibration (RMSEC) values ranged between 1.8 and 3.5, high coefficient of determination in prediction (R^2^p) values ranged between 0.82 and 0.92, and low root mean square error of prediction (RMSEP) values ranged between 2.5 and 4.3. However, prediction models developed with raw visible light spectra (400–780 nm) had a slight decrease in R^2^c values (between 0.82 and 0.87) and R^2^p values (0.68–0.83). RPD values of raw visible light spectra were less than 2.5, which indicated that the effect of raw visible light on the quantitative prediction of chemical composition of bamboo was unsatisfactory, although all RER values of visible light spectra were more than 5.6. The possible reason was that visible light only reflected surface characteristics of the material, such as color, glossiness, and light reflection, containing less information about the inner chemical composition of the material. The optimal number of factors for each model was suggested by the software Unscrambler v9.2. Based on the raw NIR spectra (780–2500 nm), regression coefficient plots of cellulose, xylan, and lignin are separately presented in Fig. 3. As shown in Fig. 3a illustrated as an example, there were distinct bands in the 1440–1480 nm region attributed to the first overtone O–H stretching vibration [29] and a remarkable peak at around 2080 nm where the C–H deformation and O–H stretching vibration of cellulose were located in the NIR spectra [28]. These wavelength regions greatly contributed to the prediction of cellulose in the suggested factor 4. Compared with PLS1 model, the results from three dependent variables modeled and predicted simultaneously (PLS2 model) were almost close to those predicted by PLS1 model in raw NIR spectra and raw visible–near infrared spectra. However, two random dependent variables modeled and predicted simultaneously presented higher coefficient of determination and lower root mean square error than three dependent variables (Additional file 1: Table S2). Considering the operating mode and efficiency, PLS2 model was better in quantitative prediction of chemical composition of bamboo.

**Table 1:** Results of PLS1 calibration and prediction models for the quantitative compositional analysis of bamboo using raw spectra

Wavelength (nm)	Chemical composition	Factors	R^2^ c	RMSEC	R^2^ p	RMSEP	SD	RPD	RER
400–780 (*n* = 36)	Cellulose	8	0.82	4.3	0.68	6.0	10.3	1.7	5.6
	Xylan	3	0.87	3.0	0.83	3.5	8.4	2.4	8.0
	Lignin	3	0.83	3.7	0.78	4.2	8.9	2.1	7.1
	Glucose	3	0.79	8.2	0.70	9.7	18.2	1.9	5.4
	Xylose	10	0.97	4.3	0.86	9.0	23.7	2.6	8.7
780–2500 (*n* = 36)	Cellulose	4	0.88	3.5	0.82	4.3	10.3	2.4	6.5
	Xylan	6	0.93	2.1	0.88	2.9	8.4	2.9	10.0
	Lignin	7	0.96	1.8	0.92	2.5	8.9	3.6	11.9
	Glucose	7	0.92	5.1	0.77	8.6	18.2	2.1	7.6
	Xylose	4	0.90	7.5	0.79	11.1	23.7	2.1	6.0
400–2500 (*n* = 36)	Cellulose	4	0.88	3.5	0.82	4.3	10.3	2.4	6.1
	Xylan	4	0.94	2.1	0.91	2.5	8.4	3.3	10.0
	Lignin	7	0.94	2.2	0.86	3.3	8.9	2.7	8.7
	Glucose	4	0.84	7.1	0.73	9.4	18.2	1.9	5.9
	Xylose	5	0.87	8.4	0.75	11.7	23.7	2.0	7.0

*R* ^*2*^ *c* square of the correlation coefficient for calibration, *RMSEC* root mean square error of calibration, *R* ^*2*^ *p* square of the correlation coefficient for prediction, *RMSEP* root mean square error of prediction, *SD* standard deviation, *RPD* ratio of root mean square error of prediction to standard deviation, *RER* range error ratio. The number of samples used for quantitative analysis of cellulose, xylan, and lignin is 36. The number of samples used for quantitative analysis of glucose and xylose is 26

**Fig. 4:**
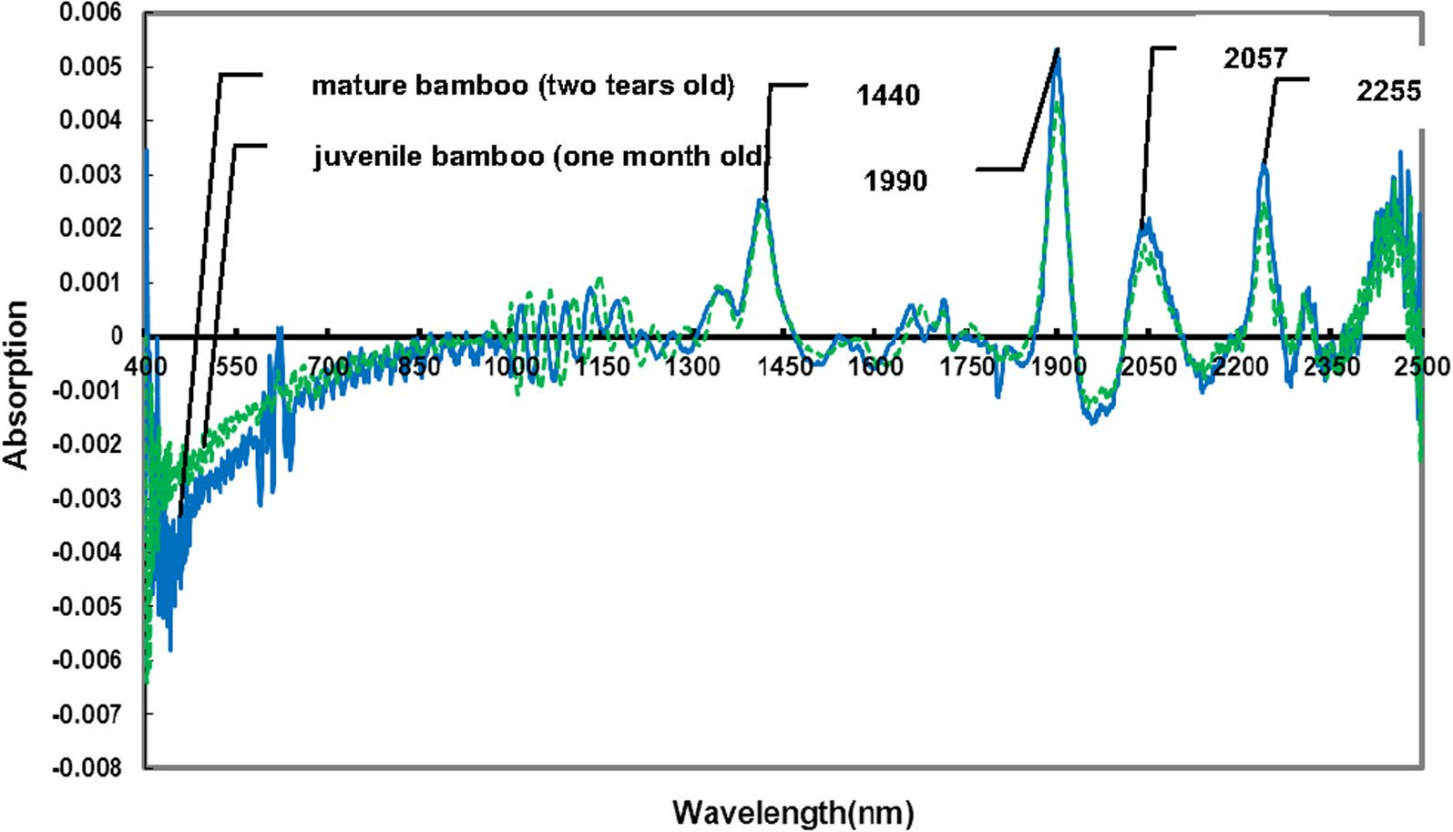
First derivative visible–near infrared spectra of bamboo timber fraction. Data of one spectrum were collected from 2-year-old mature bamboo and those of another spectrum were collected from one-month-old juvenile bamboo

Several data pretreatment methods, including multiplicative scattering correction (MSC), extensive multiplicative scattering correction (EMSC), standard normalized variate (SNV), first derivative, and second derivative pretreatment, were tested on raw visible–near infrared spectra. The first derivative visible–near infrared spectra of bamboo timber fraction of one-month-old juvenile bamboo and 2-year-old mature bamboo are presented in Fig. 4. The pretreated spectra were mainly dominated by the peaks at around 1440, 1900, 2057, and 2255 nm. The peak at around 1440 nm corresponded to the first overtone O–H stretching in cellulose [30]. The absorbance at approximately 1907 nm was assigned to the second overtone of *C* = O stretching in xylan. The bands near 2057 nm were associated with C–H deformation and O–H stretching vibration. The peak at 2255 nm was attributed to the overtone of O–H stretching and C-O stretching vibration [28].

The predicted chemical contents (cellulose, xylan, and lignin) via first derivative pretreated visible–near infrared spectra vs. wet chemistry measurements as results generated by the PLS regression procedure are presented in Fig. 2b. Compared with raw visible–near infrared spectra, the correlation was higher between the predicted and actual values. Results of PLS1 and PLS2 calibration and prediction models for the quantitative compositional analysis of bamboo using pretreated visible–near infrared spectra are presented in Table 2 and Additional file 1: Table S3. In Table 2, compared with other methods, first and second derivative pretreatments mainly showed higher R^2^c and R^2^p (ranged from 0.990 to 0.996 and 0.976 to 0.994, respectively), lower RMSEC and RMSEP (ranged from 0.5 to 1.1 and 0.7 to 1.5, respectively), and higher RPD and RER (ranged from 8.3 to 10.2 and 20.3 to 35.2, respectively). Additional file 1: Table S3 demonstrates the same trend of results of PLS2 models with PLS1 models. The results showed that first derivative and second derivative pretreatments were relatively better pretreatment methods to clearly improve the accuracies of the prediction performance of both PLS1 and PLS2 models in this study.

**Table 2:** Results of PLS1 calibration and prediction models for the quantitative compositional analysis of bamboo using pretreated visible–near infrared spectra

Pretreatment	Chemical composition	Factors	R^2^ c	RMSEC	R^2^ p	RMSEP	SD	RPD	RER
MSC	Cellulose	10	0.958	2.1	0.925	2.8	10.0	3.6	11.8
	Xylan	3	0.947	1.9	0.931	2.2	8.1	3.7	12.2
	Lignin	8	0.972	1.5	0.939	2.2	8.8	4.1	13.3
	Glucose	16	0.998	0.7	0.986	2.2	18.2	8.4	26.4
	Xylose	14	0.998	1.3	0.974	3.8	23.7	6.3	17.5
EMSC	Cellulose	10	0.964	1.9	0.935	2.6	10.1	3.9	12.5
	Xylan	3	0.953	1.8	0.937	2.1	8.2	3.9	13.0
	Lignin	7	0.964	1.7	0.943	2.1	8.7	4.2	13.3
	Glucose	16	0.998	0.4	0.992	1.6	18.2	11.2	37.7
	Xylose	14	0.998	1.1	0.980	3.3	23.7	7.3	20.5
SNV	Cellulose	5	0.910	3.0	0.874	3.6	9.8	2.7	7.9
	Xylan	4	0.955	1.8	0.935	2.1	8.2	3.9	13.3
	Lignin	8	0.974	1.4	0.949	2.0	8.8	4.3	13.8
	Glucose	15	0.996	1.1	0.974	2.9	18.2	6.3	21.0
	Xylose	15	0.998	1.0	0.980	3.3	23.7	7.2	20.0
First derivative	Cellulose	8	0.994	0.8	0.990	1.0	10.2	10.1	35.2
	Xylan	7	0.990	0.8	0.982	1.1	8.3	7.7	25.2
	Lignin	7	0.988	1.0	0.980	1.2	8.8	7.2	22.7
	Glucose	9	0.998	0.6	0.996	1.1	18.2	16.1	52.6
	Xylose	7	0.998	1.1	0.994	1.8	23.7	13.0	34.3
Second derivative	Cellulose	7	0.988	1.1	0.976	1.5	10.2	6.6	20.3
	Xylan	7	0.990	0.8	0.984	1.0	8.3	8.1	29.0
	Lignin	10	0.996	0.5	0.994	0.7	8.9	13.7	36.6
	Glucose	8	0.998	0.7	0.996	1.1	18.2	16.8	51.8
	Xylose	7	0.996	1.4	0.992	2.0	23.7	11.7	32.5

*R* ^*2*^ *c* square of the correlation coefficient for calibration, *RMSEC* root mean square error of calibration, *R* ^*2*^ *p* square of the correlation coefficient for prediction, *RMSEP* root mean square error of prediction, *SD* standard deviation, *RPD* ratio of root mean square error of prediction to standard deviation, *RER* range error ratio. *MSC* multiplicative scattering correction, *EMSC* extensive multiplicative scattering correction, *SNV* standard normalized variate. The number of samples used for quantitative analysis of cellulose, xylan, and lignin is 36. The number of samples used for quantitative analysis of glucose and xylose is 26

Similarly, the general enzymatic digestibilities of the bamboo fractions were forecasted by means of glucose and xylose yields. The plots comparing the predicted and actual values were generated to show visually the prediction performance of the model in raw visible–near infrared spectra (Fig. 2c). The experimental values and estimated values for glucose and xylose are presented in Additional file 1: Table S4. The result indicated that the prediction performance of the model was not very well. Results of PLS1 and PLS2 calibration and prediction models for the quantitative sugars of enzymatic hydrolysis analysis of bamboo using raw spectra (visible light, NIR and visible–near infrared) are presented in Table 1 and Additional file 1: Table S2, respectively. Low RMSEC values (ranged between 4.3 and 8.4), RMSEP values (ranged between 8.6 and 11.8), RPD values (low to 1.8), and RER values (low to 5.4) were observed, indicating that the performance of PLS1 and PLS2 models was not well in quantitatively analyzing glucose and xylose yields of bamboo using raw spectra. The possible reasons for the phenomenon were that the original spectra contained more noise, the number of samples was small, the changes of sugar content in samples were slight, and the representation of the sample for modeling was slightly worse. Possibly, increasing the number of samples and the representation of the sample could improve the results in the further study.

Raw visible–near infrared spectra were pretreated by the five pretreatment methods. The plots comparing the predicted and actual values were generated to show visually the prediction performance of the model in first derivative pretreated visible–near infrared spectra (Fig. 2d). Compared with raw spectra, first derivative pretreatment significantly improved the correlation between the predicted and actual values. Results of calibration and prediction in PLS1 and PLS2 models for the quantitative sugars of enzymatic hydrolysis of bamboo using pretreated visible–near infrared spectra are presented in Table 2 and Additional file 1: Table S3, respectively. Compared with raw visible–near infrared spectra, both R^2^c values (ranged from 0.988 to 0.998) and R^2^p values (ranged from 0.968 to 0.996) were significantly improved, and both RPD values (more than three) and RER values (more than 15) also greatly verified the better performance of PLS1 and PLS2 models. The results showed that the pretreatment could greatly reduce the noise and improve the signal-to-noise ratio, so that the performance of the PLS1 and PLS2 models was greatly improved. Hence, pretreated visible–near infrared spectra coupled with PLS regression was able to quantitatively predict general hydrolysabilities of bamboo fractions after pretreatment with aqueous ammonia.

### Qualitative classification of bamboo fractions

**Fig. 5:**
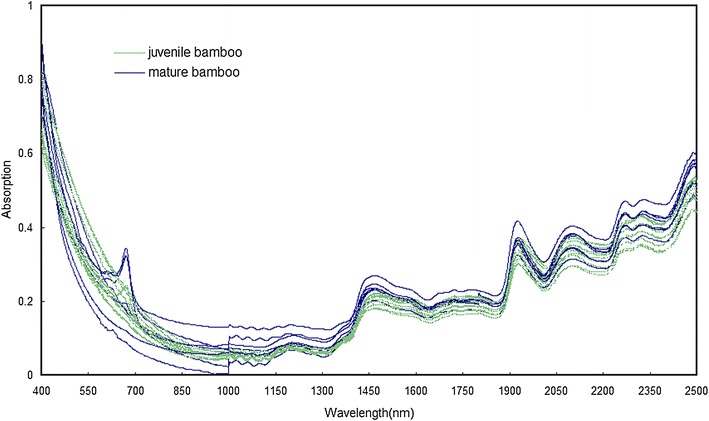
Visible–near infrared mean spectra from original data of bamboos. Ten spectra were collected from 2- and 10-year-old mature bamboo and other 10 spectra were collected from 2-, 4- and 6-meter-height juvenile bamboo

There existed differences in processing residues between mature bamboo and juvenile bamboo. If mature bamboo and juvenile bamboo could be quickly and accurately discriminated, these materials would reasonably optimize utilization. The mean spectra from original data for mature bamboo fractions and juvenile bamboo fractions between 400 and 2500 nm are presented in Fig. 5. A notable peak occurred in the wavelength region of 600–700 nm, which was generated by samples of bamboo branch and bamboo green. The reason may be that chlorophyll a provided by bamboo branch and bamboo green caused the peak formation. As shown in Fig. 5, the spectra of mature bamboo basically had higher absorbance value because of different contents of chemical composition in mature bamboo and juvenile bamboo. For example, the content of lignin in mature bamboo was higher than that in juvenile bamboo.

**Fig. 6:**
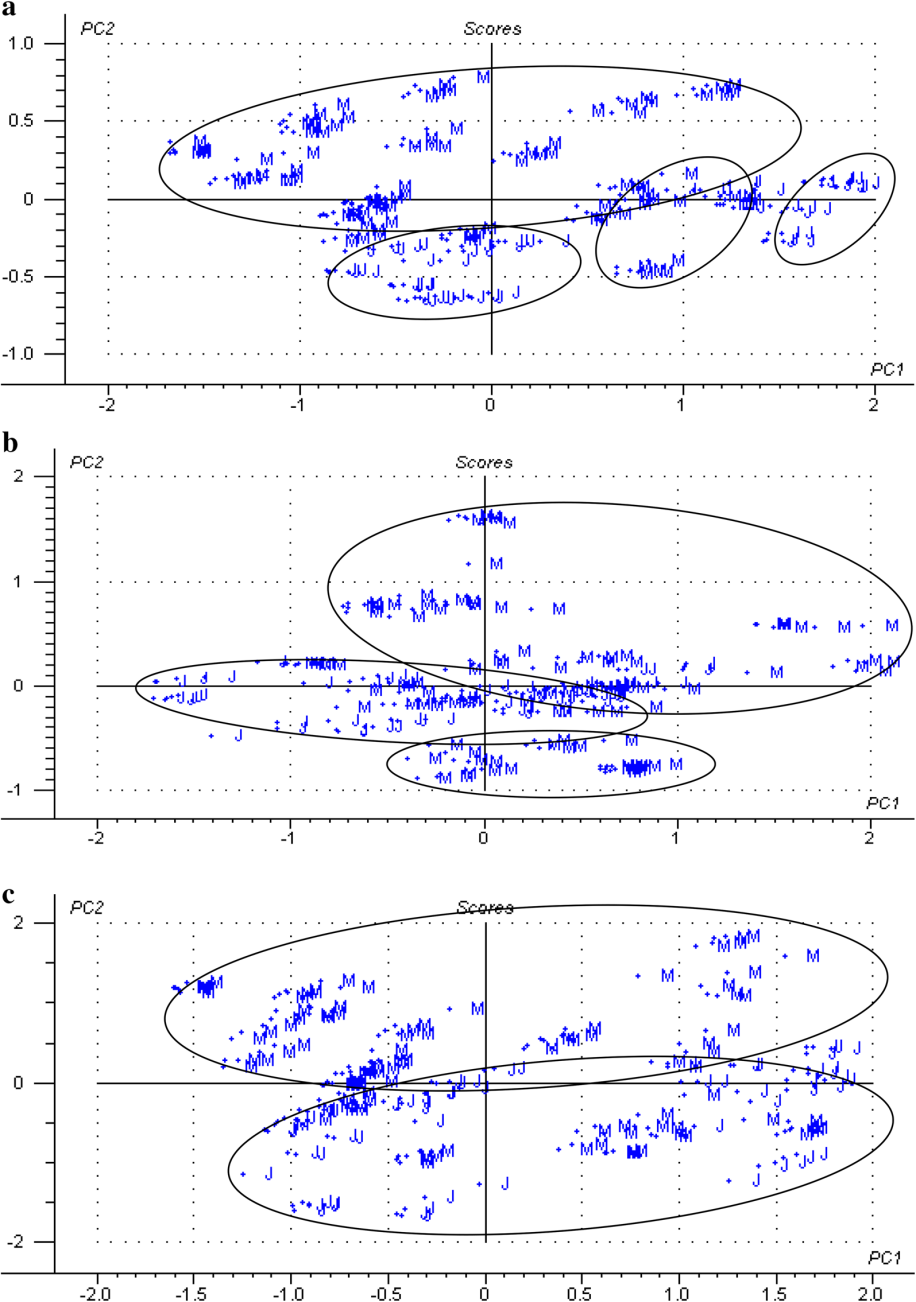
PCA analysis of mature bamboo and juvenile bamboo based on different raw spectral regions: **a** raw visible light spectral region; **b** raw NIR spectral region; and **c** raw visible–near infrared spectral region. (M—mature bamboo; J—juvenile bamboo)

**Fig. 7:**
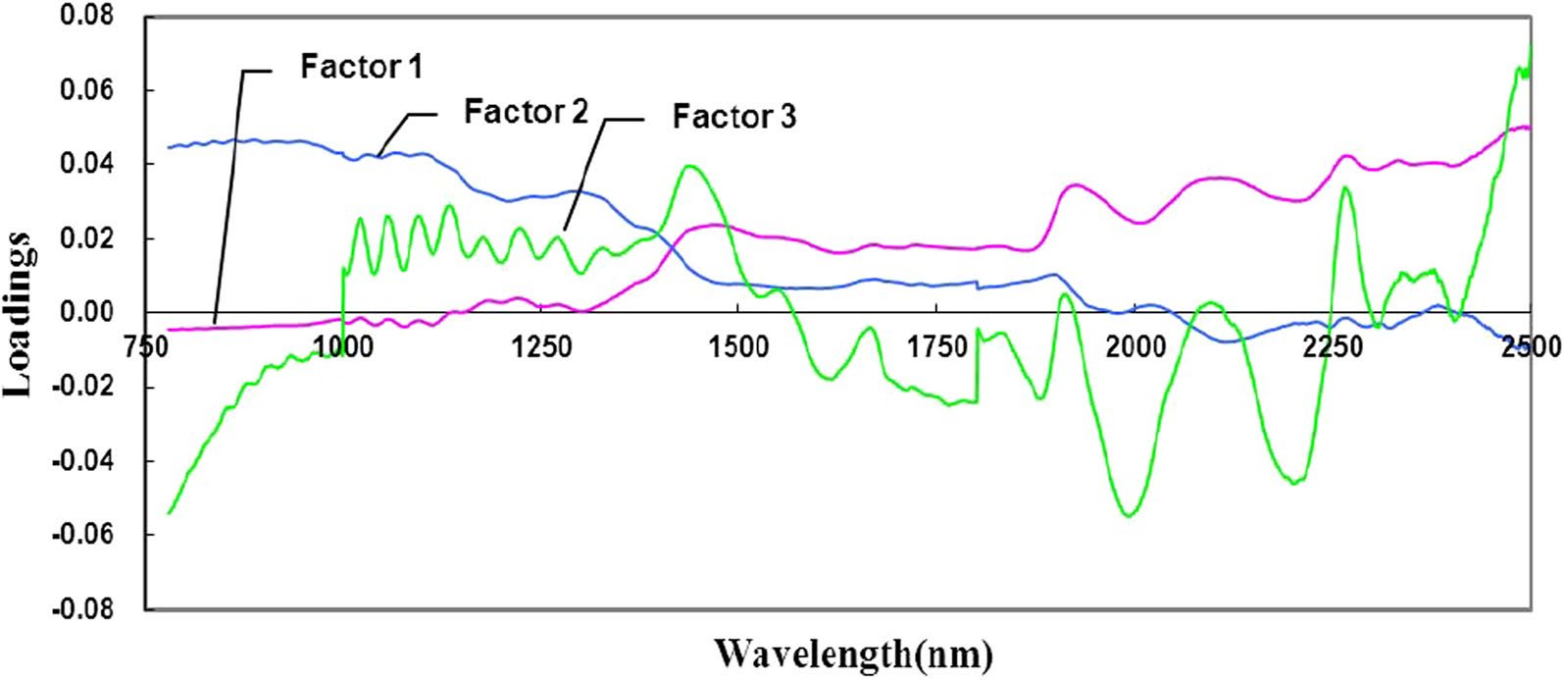
Loading plots of the first three factors of the raw near infrared spectra. Factor 1 has a proportion of variance of 64 %; factor 2 has a proportion of variance of 28 %; and factor 3 has a proportion of variance of 6 %

The results of principal component analysis (PCA) of mature bamboo and juvenile bamboo using raw spectra (visible light, NIR, and visible–near infrared spectra) were obtained (Fig. 6). It was evident from these three PCA score plots that mature bamboo could be separated from juvenile bamboo with 95 % confidence. As shown in Fig. 6a, c, the distribution of most mature bamboo samples was higher than that of juvenile bamboo samples in the coordinate axis. And the distribution of the most mature bamboo samples preferred the offside in Fig. 6b. Loading plots of the first three factors of the raw near infrared spectra are shown in Fig. 7. The first three factors had a greater contribution to principal component analysis in the large wavelength range (1100–2500 nm) than in the short wavelength range (780–1100 nm), especially spectral bands in the 1420–1470 nm and 1870–2300 nm regions were more remarkable. From the point of view of factor 1, there existed significant peaks at around 1448, 1930, and 2110 nm, which were separately associated with the first overtone O–H stretching, the O–H asymmetric stretching, and O–H deformation from water, and the C–H deformation and O–H stretching in cellulose. For factor 2, the main absorptions at 1440 nm and 2267 nm were related to the first overtone O–H stretching and O–H and C-O stretching in lignin, respectively. For factor 3, the peak at around 1907 nm was attributed to the second overtone of *C* = O stretching in xylan. The results of the prediction models were good, exhibiting high R^2^c values (ranged between 0.96 and 0.98) and R^2^p values (ranged between 0.94 and 0.97), and low square error of calibration (SEC) values (ranged between 0.07 and 0.10) and square error of validation (SEV) values (ranged between 0.09 and 0.12) (Table 3). Then PLS-DA identification models based on three different wavelength regions were established, aiming to test the ability and accuracy of NIR models. Based on PLS-DA, results of the unknown samples of mature bamboo and juvenile bamboo predicted by identification models are presented in Table 4. The classification accuracies of mature bamboo and juvenile bamboo from the prediction set using the model based on samples from the corresponding calibration set all reached 100 %. It indicated that the PLS-DA models had the ability to quickly predict and classify mature bamboo and juvenile bamboo.

**Table 3:** Results of calibration and prediction models for discriminating mature bamboo and juvenile bamboo

Wavelength (nm)	Sample sets	Factors	*R* ^2^	SEC/SEV	Factors	*R* ^2^	SEC/SEV
400–780 (*n* = 160)	Calibration	10	0.96	0.10	10	0.96	0.10
	Validation		0.94	0.12		0.94	0.12
780–2500 (*n* = 160)	Calibration	7	0.98	0.08	7	0.98	0.08
	Validation		0.96	0.09		0.96	0.09
400–2500 (*n* = 160)	Calibration	9	0.98	0.07	9	0.98	0.07
	Validation		0.97	0.09		0.97	0.09

Three regions of wavelength were selected to establish models, and these spectra came from original data. The number of juvenile bamboo and mature bamboo samples were 60 and 100, respectively

**Table 4:** Results of juvenile bamboo and mature bamboo predicted by identification models based on PLS-DA

Wavelength (nm)	Type	No. of prediction samples	Correct no.	Accuracy (%)
400–780 (*n* = 160)	Juvenile bamboo	24	24	100
	Mature bamboo	40	40	100
780–2500 (*n* = 160)	Juvenile bamboo	24	24	100
	Mature bamboo	40	40	100
400–2500 (*n* = 160)	Juvenile bamboo	24	24	100
	Mature bamboo	40	40	100

Three regions of wavelength were selected to establish models, and these spectra came from original data. The number of juvenile bamboo and mature bamboo samples were 60 and 100, respectively

**Fig. 8:**
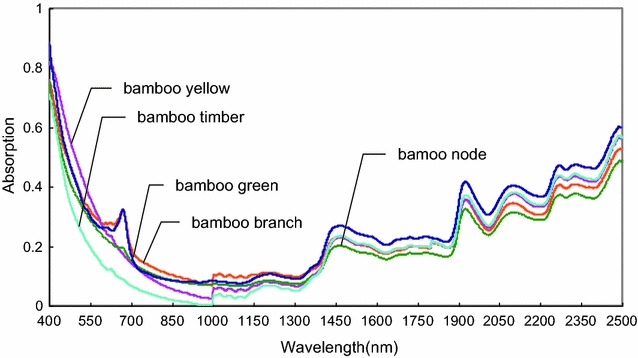
Visible–near infrared mean spectra from original data of different fractions. The five spectra were collected from bamboo node, bamboo branch, bamboo green, bamboo timber, and bamboo yellow of 2-year-old bamboo, respectively

Similarly, different bamboo fractions were qualitatively classified using PLS-DA models. The visible–near infrared spectra of different fractions of 2-year-old bamboo samples are shown in Fig. 8. Spectral lines of bamboo yellow and bamboo timber were almost overlapped in 2000–2500 nm range. Probably because the three peaks (2092, 2267, and 2238 nm) in the wavelength region mainly reflected the information on xylan and lignin, the contents of which in bamboo yellow and bamboo timber were very close. The gaps of spectral lines in other fractions were obvious.

**Fig. 9:**
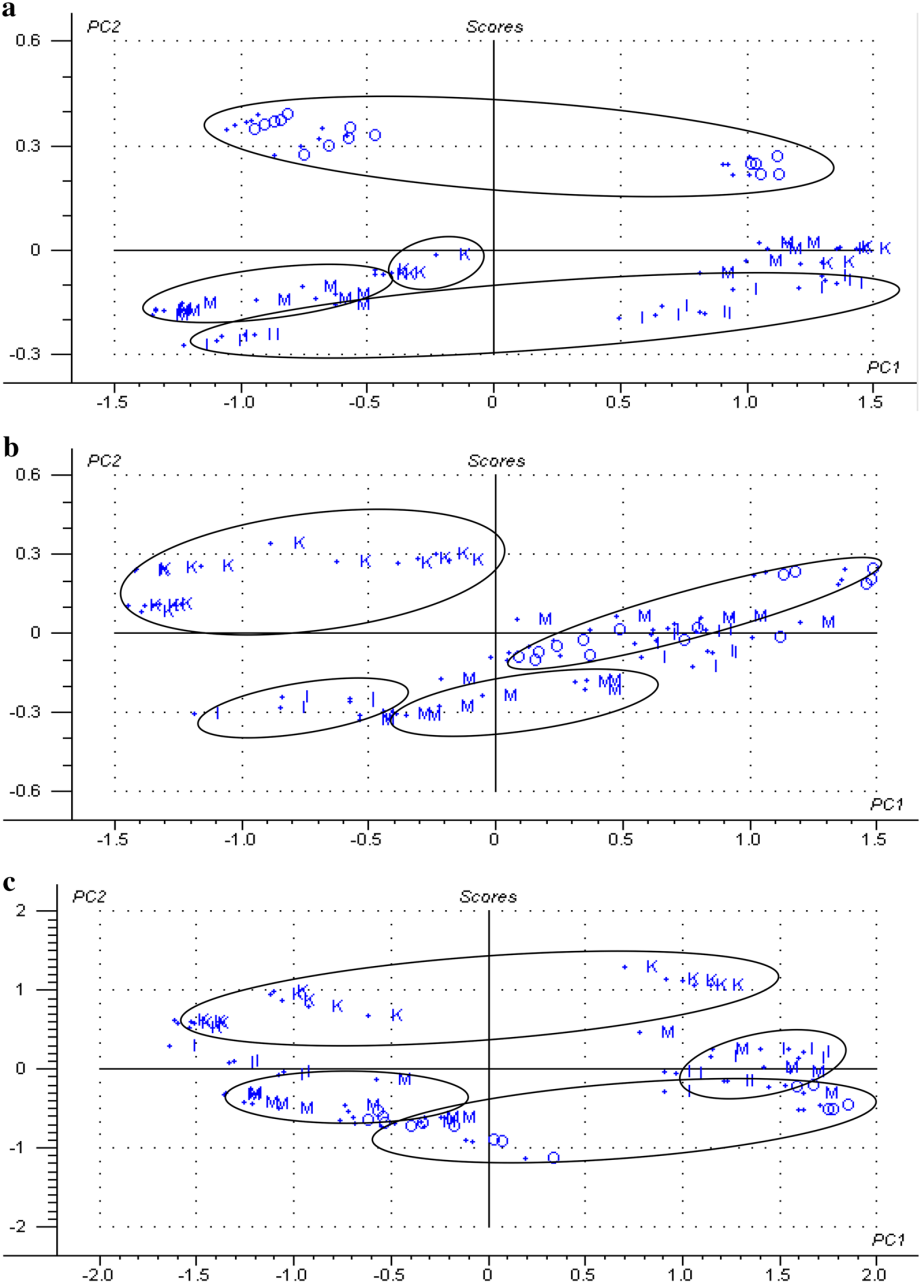
PCA analysis of juvenile bamboo fractions based on different raw spectral regions: **a** raw visible light spectral region; **b** raw NIR spectral region; and **c** raw visible–near infrared spectral region. (O—bamboo green; M—bamboo timber; I—bamboo yellow; K—bamboo node)

The results of PCA analysis of juvenile bamboo fractions using raw spectra (visible light, NIR, and visible–near infrared spectra) were obtained (Fig. 9). There were some score plots of samples in three different spectral regions assigned the wrong groups basically. For example, some samples of bamboo yellow were confused with samples of bamboo node in visible light spectra (Fig. 9a), and samples of bamboo green, bamboo yellow, and bamboo timber were confused together in NIR spectra and visible–near infrared spectra (Fig. 9b, c). The possible reason was that the contents of chemical composition in juvenile bamboo fractions were close. Therefore, it was not reasonable to identify different fractions using samples of juvenile bamboo.

**Fig. 10:**
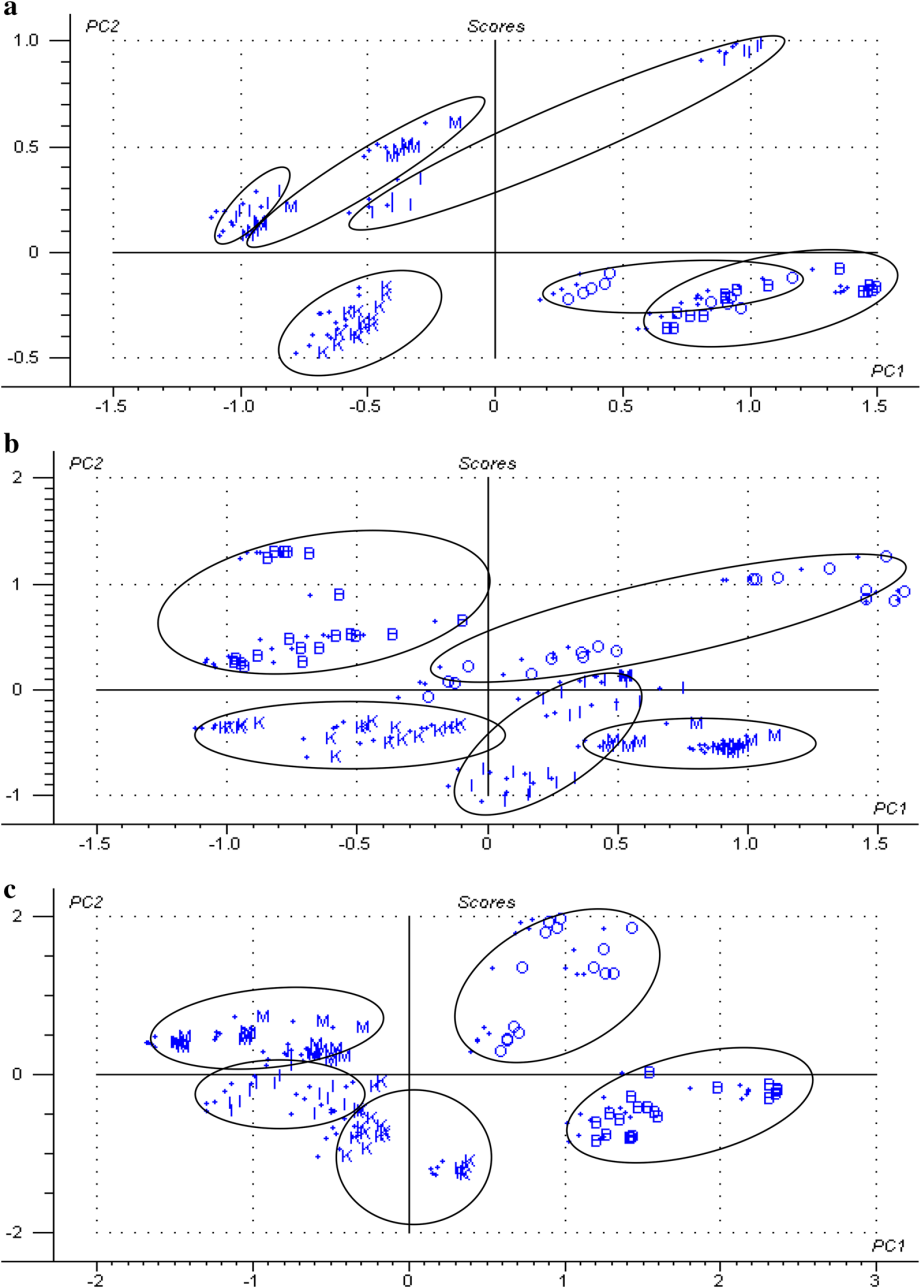
PCA analysis of mature bamboo fractions based on different raw spectral regions: **a** raw visible light spectral region; **b** raw NIR spectral region; and **c** raw visible–near infrared spectral region. (O—bamboo green; M—bamboo timber; I—bamboo yellow; K—bamboo node; B—bamboo branch)

The results of PCA analysis of mature bamboo fractions using raw spectra (visible light, NIR, and visible–near infrared spectra) were obtained (Fig. 10). The score plots of different fraction samples in the three spectral regions were obviously distinguished, except for some samples of bamboo green confused with samples of bamboo node and branch in visible light spectra (Fig. 7a). The results of the prediction models were good, exhibiting high R^2^c values ranged between 0.81 and 0.98, low SEC values ranged between 0.05 and 0.17, high R^2^p values ranged between 0.69 and 0.95, and low SEV values ranged between 0.09 and 0.23 (Table 5). Based on PLS-DA, results of the unknown sample of mature bamboo fractions predicted by identification models are presented in Table 6. The classification accuracies of different fractions of mature bamboo from the prediction set using the model based on samples from the corresponding calibration set all reached 100 %, except for visible light spectral range where one sample of the bamboo knot was misclassified into other bamboo fractions. However, the predictive performance of this model still presented a high total prediction accuracy of 87.5 %.

**Table 5:** Results of calibration and prediction models for classifying mature bamboo fractions

Wavelength (nm)	Sample sets	Parameters	B	I	K	M	O
400–780 (*n* = 100)	Calibration	*R* ^2^	0.96	0.88	0.81	0.93	0.93
		SEC	0.08	0.14	0.17	0.11	0.11
	Validation	*R* ^2^	0.90	0.77	0.69	0.71	0.83
		SEV	0.13	0.19	0.23	0.21	0.17
	Factors		13	13	13	13	13
780–2500 (*n* = 100)	Calibration	*R* ^2^	0.98	0.94	0.93	0.97	0.97
		SEC	0.05	0.10	0.10	0.08	0.07
	Validation	*R* ^2^	0.93	0.76	0.76	0.85	0.90
		SEV	0.11	0.20	0.20	0.16	0.13
	Factors		16	16	16	16	16
400–2500 (*n* = 100)	Calibration	*R* ^2^	0.98	0.98	0.96	0.97	0.96
		SEC	0.06	0.06	0.08	0.07	0.08
	Validation	*R* ^2^	0.94	0.95	0.87	0.90	0.91
		SEV	0.10	0.09	0.14	0.13	0.12
	Factors		15	15	15	15	15

Three regions of wavelength were selected to establish models, and these spectra came from original data. The number of sample sets was 100. (*B* bamboo branch, *I* bamboo yellow, *K* bamboo node, *M* bamboo timber, *O* bamboo green)

**Table 6:** Results of mature bamboo fractions predicted by identification models based on PLS-DA

Wavelength (nm)	Fractions	No. of prediction samples	Correct no.	Accuracy (%)
400–780 (*n* = 100)	B	8	8	100
	I	8	8	100
	K	8	7	87.50
	M	8	8	100
	O	8	8	100
780–2500 (*n* = 100)	B	8	8	100
	I	8	8	100
	K	8	8	100
	M	8	8	100
	O	8	8	100
400–2500 (*n* = 100)	B	8	8	100
	I	8	8	100
	K	8	8	100
	M	8	8	100
	O	8	8	100

Three regions of wavelength were selected to establish models, and these spectra came from original data. The number of sample sets was 100. (*B* bamboo branch, *I* bamboo yellow, *K* bamboo node, *M* bamboo timber, *O* bamboo green)

### Qualitative analysis of the sugar yield level

4- and 6-meter-height juvenile bamboos were hydrolyzed with cellulases and xylanase to evaluate the enzymatic digestibilities of the materials. In the study, dividing line between high and low levels of the glucose content was artificially set to 70, and dividing line between high and low levels of the xylose content was artificially set to 40 in order to qualitatively analyze the sugar content level. Three-fifth of the samples were randomly selected from high and low sugar content samples to establish calibration models, and the remaining two-fifth of the samples were used for predictions. The results of the prediction models were good, exhibiting high R^2^c values ranged between 0.939 and 0.998, low SEC values ranged between 0.01 and 0.13, high R^2^p values ranged between 0.491 and 0.891, and low SEV values ranged between 0.17 and 0.38 (Table 7). Based on PLS-DA, the results of unknown samples of glucose and xylose content level in 4-meter-height and 6-meter-height juvenile bamboo dealt with cellulases and hemicellulases before alkaline pretreatment are presented in Table 8. The classification accuracies of high and low levels of glucose and xylose content of unknown samples were all 100 %.

**Table 7:** Results of calibration and prediction models for analysis of glucose and xylose content level

Wavelength (nm)	Sample sets	Level	Before aqueous ammonia pretreatment (*n* = 40)				After aqueous ammonia pretreatment (*n* = 90)			
			Glucose		Xylose		Glucose		Xylose	
			*R* ^2^	SEC/SEV	*R* ^2^	SEC/SEV	*R* ^2^	SEC/SEV	*R* ^2^	SEC/SEV
400–780	Calibration	High	0.998	0.01	0.996	0.03	0.821	0.21	0.941	0.12
		Low	0.998	0.01	0.996	0.03	0.821	0.21	0.941	0.12
	Validation	High	0.743	0.26	0.808	0.23	0.814	0.22	0.869	0.17
		Low	0.743	0.26	0.808	0.23	0.814	0.22	0.869	0.17
	Factors	High	11		9		1		8	
		Low	11		9		1		8	
780–2500	Calibration	High	0.955	0.11	0.978	0.08	0.810	0.22	0.850	0.19
		Low	0.955	0.11	0.978	0.08	0.810	0.22	0.850	0.19
	Validation	High	0.491	0.38	0.857	0.20	0.783	0.24	0.783	0.22
		Low	0.491	0.38	0.857	0.20	0.783	0.24	0.783	0.22
	Factors	High	7		7		3		6	
		Low	7		7		3		6	
400–2500	Calibration	High	0.939	0.13	0.984	0.07	0.994	0.04	0.978	0.07
		Low	0.939	0.13	0.984	0.07	0.994	0.04	0.978	0.07
	Validation	High	0.748	0.26	0.891	0.17	0.953	0.11	0.924	0.13
		Low	0.750	0.26	0.891	0.17	0.953	0.11	0.924	0.13
	Factors	High	7		8		12		11	
		Low	7		8		12		11	

The data of glucose and xylose content level and spectra were collected from 4- and 6-meter-height juvenile bamboo before aqueous ammonia pretreatment, and 2- and 10-year-old mature bamboo and 4- and 6-meter-height juvenile bamboo after aqueous ammonia pretreatment

**Table 8:** Prediction results of unknown samples of glucose and xylose content level

Wavelength (nm)	Sugars	Level	Before aqueous ammonia pretreatment (*n* = 40)			After aqueous ammonia pretreatment (*n* = 90)		
			No. of prediction samples	Correct no.	Accuracy (%)	No. of prediction samples	Correct no.	Accuracy (%)
400–780	Glucose	High	10	10	100	18	18	100
		Low	6	6	100	18	18	100
	Xylose	High	8	8	100	24	24	100
		Low	8	8	100	12	12	100
780–2500	Glucose	High	10	10	100	18	18	100
		Low	6	6	100	18	18	100
	Xylose	High	8	8	100	24	24	100
		Low	8	8	100	12	12	100
400–2500	Glucose	High	10	10	100	18	18	100
		Low	6	6	100	18	18	100
	Xylose	High	8	8	100	24	24	100
		Low	8	8	100	12	12	100

The data of glucose and xylose content level and spectra were collected from 4- and 6-meter-height juvenile bamboo before aqueous ammonia pretreatment, and 2- and 10-year-old mature bamboo and 4- and 6-meter-height juvenile bamboo after aqueous ammonia pretreatment (based on PLS-DA)

2- and 10-year-old mature bamboo and 4- and 6-meter-height juvenile bamboo after pretreatment with aqueous ammonia were hydrolyzed with cellulases and xylanase, and the amounts of glucose and xylose released were evaluated. In the study, dividing lines between high and low levels of the glucose content and xylose content were both set to 80. Three-fifth of the samples were randomly selected from high and low sugar content samples to establish calibration models, and the remaining two-fifth of the samples were used for predictions. The results of the prediction models were good, exhibiting high R^2^c values ranged between 0.810 and 0.994, low SEC values ranged between 0.04 and 0.22, high R^2^p values ranged between 0.783 and 0.953, and low SEV values ranged between 0.11 and 0.24 (Table 7). Based on PLS-DA, the results of unknown samples of glucose and xylose content level in 2- and 10-year-old and 4- and 6-meter-height juvenile bamboo dealt with cellulases and xylanase after alkaline pretreatment are presented in Table 8. The classification accuracies of high and low levels of glucose and xylose contents of unknown samples were all 100 %.

## Conclusions

Visible–near infrared spectroscopy coupled with multivariate analysis was applied to quantitatively analyze the chemical composition of bamboo and general hydrolysabilities of juvenile bamboo and bamboo after pretreatment with aqueous ammonia, and qualitatively discriminate between mature bamboo and juvenile bamboo. The results indicated that PLS regression method had the potential to quantitatively analyze the chemical composition and the enzymatic digestibilities of bamboo fractions. Considering the operating mode and efficiency, PLS2 model was better in quantitative prediction of chemical composition of bamboo and the PLS-DA models had the ability to predict and classify mature bamboo, juvenile bamboo, and different fractions of mature bamboo.

## Methods

### Sample preparation

Moso bamboo (*Phyllostachys heterocycla* var. pubescens) samples including 2-, 4-, 6-, and 10-year-old mature bamboos and 2-, 4-, and 6-meter-height juvenile bamboos were collected from a bamboo plantation located in Zhejiang Province, China. The 2-, 4-, 6-, and 10-year-old mature bamboos are about 11, 15, 13, and 15 meter in height, respectively. For 2-, 4-, and 6- meter-height juvenile bamboos, they are about one month old. Mature bamboos were fractionated manually with a knife to five parts: bamboo green, timber, yellow, node, and branch (the part where juvenile bamboo do not exist). All the bamboo fractions were milled and passed through 60-mesh screen sieve, and then air dried to less than 10 % moisture content. The materials were pretreated with 26 % (w/v) aqueous ammonia with a solid-to-liquid ratio of 1:10 at 70 °C for 72 h. The pretreated bamboo fractions were washed to neutral with pure water and then air dried for further use. Chemical composition of the materials before and after pretreatment with aqueous ammonia was determined according to the standardized methods established by the National Renewable Energy Laboratory [31]. The raw juvenile bamboos and aqueous ammonia-pretreated juvenile and mature bamboo fractions were hydrolyzed by cellulases (20 FPU/g dry matter Celluclast 1.5 L and 500 nkat/g dry matter Novozyme 188) and xylanase (2 mg/g dry matter) for 48 h. The amount of glucose and xylose released was evaluated by an HPLC system (Hitachi L-2000, Hitachi Corp., Japan). The system was equipped with a refractive index detector (Hitachi Corp., Japan) and an autosampler (Hitachi Corp., Japan). Ion-moderated partition chromatography column (Aminex column HPX-87H) with Cation H micro-guard cartridge was used. The Aminex HPX-87H column was maintained at 45 °C with 5 mM H_2_SO_4_ as the eluent at a flow rate of 0.5 ml/min. Before injection, samples were filtered through 0.22 µm MicroPES filters, and a volume of 20 µl was injected. Peaks were detected by refractive index and were indentified and quantified by comparison to retention times of authentic standards (d-glucose and D-xylose).

### NIR spectral data acquisition

NIR diffuse reflectance spectrum (350–2500 nm) was collected using the ASD Field Spec^®^ NIR spectrometer (Analytical Spectral Devices, Boulder, CO, USA) at room temperature. A fiber optic probe was oriented perpendicular to the sample surface and used to collect spectra. The instrument reference was a piece of commercial microporous^®^ Teflon white board. Thirty scans were recorded and the results were averaged to yield the final spectrum. All spectroscopy measurements were made in a controlled humidity chamber (50–60 %) and at 20 ± 2 °C. Three spectral segments (400–780, 780–2500, and 400–2500 nm) were selected in the study for comprehensive analysis of different spectral ranges.

### Multivariate analysis (MVA)

The MVA of NIR spectra for qualitative classification and quantitative chemical composition prediction was conducted using the software Unscrambler v9.2 (CAMO, Corvallis, OR, USA). In terms of quantitative chemical composition, MSC, EMSC, SNV, first derivative and second derivative of spectral range between 400 and 2500 nm were also analyzed to compare with raw spectra. PLS regression analysis was calculated to determine the quantitative relation between the spectral variable and the chemical composition content of the samples. PLS is a linear modeling method compressing the spectral data and projecting them onto partial least squares components, which can be divided into the PLS1 and PLS2 methods. The PLS1 method extracts the spectral information into the PLS components to ensure the maximized covariance to the dependent variable. In the PLS2 method, two or more dependent variables are modeled simultaneously. The regression coefficient plots were used to analyze PLS models for each composition [32]. The accuracy of the model was evaluated by the determination of R^2^c, RMSEC, R^2^p, RMSEP RPD, and RER (Range_y_ of reference data/standard error of prediction (SEP)). The optimal number of PLS principle components was suggested by the software Unscrambler v9.2. RPD statistic was particularly applied to evaluate the prediction abilities between alternative models. RPD was calculated using the following equation:

$$RPD = \frac{SD}{RMSEP}.$$

A summary of previous studies using RPD suggests that a model with an RPD value of less than 2.5 is not able to provide sufficient prediction, whereas a model with RPD value in the range of 2.5–3 and more than three provides good and excellent prediction, respectively [33]. According to the American Association of Cereal Chemists (AACC) Method 39-00 [34], any model that has RER ≥4 is qualified for screening calibration. When RER ≥10, the model is acceptable for quality control, and if RER ≥15 the model is very good for research quantification [35].

On the other hand, prior to qualitative classification based on NIR spectra combined with PLS-DA, an individual PCA supervised the specific class-belonging of all the training set objects in advance. PCA is a multivariate method that can estimate the correlation structure of the variables. It can reduce the dimensionality of the original variables according to the importance of a variable in a PC model [36]. Samples from prediction set were selected for discriminant analysis using the PLS-DA models. PLS-DA involves developing a conventional PLS regression model, in which the variable is a binary variable. If a variable takes the value of 1, the specimen in question is a member of that group and if a variable takes the value of 0, the specimen in question is not a member of that group. In the predicted results, samples with Y variable (predicted category variable) >0.5 and a deviation that does not cross the 0.5 line would belong to the group and samples with Y variable (predicted category variable) <0.5 and a deviation that does not cross the 0.5 line would not belong to the group, and samples with a deviation that crosses the 0.5 line could not be safely recognized [37]. About three-fifth of the samples were used as calibration set, and the rest of the samples were used for predictions.

## Additional file

10.1186/s13068-016-0443-z The experimental values and estimated values for cellulose, xylan and lignin. **Table S2.** Results of calibration and prediction PLS2 models for the quantitative compositional analysis of bamboo using raw spectra. **Table S3.** Results of calibration and prediction PLS1 models for the quantitative compositional analysis of bamboo using pretreated visible-near infrared spectra. **Table S4.** The experimental values and estimated values for glucose and xylose.

## Abbreviations

EMSC: extensive multiplicative scattering correction
HPLC: high-performance liquid chromatograph
MVA: multivariate analysis
NIR: near infrared spectroscopy
PCA: principal component analysis
PLS: partial least squares
PLS-DA: partial least squares discriminant analysis
R^2^c: coefficient of determination in calibration
RMSEC: root mean square error of calibration
RMSEP: root mean square error of prediction
R^2^p: coefficient of determination in prediction
RPD: ratio of RMSEP to standard deviation
RER: range error ratio
SNV: standard normalized variate
