# Supplementary material for: Rapid determination of chemical composition and classification of bamboo fractions using visible–near infrared spectroscopy coupled with multivariate data analysis
Source: Biotechnol Biofuels. 2016 Feb 9;9:35. doi: 10.1186/s13068-016-0443-z (PMC4746775; doi:10.1186/s13068-016-0443-z)
Supplement: Supplementary file 1 — 10.1186/s13068-016-0443-z The experimental values and estimated values for cellulose, xylan and lignin. Table S2. Results of calibration and prediction PLS2 models for the quantitative compositional analysis of bamboo using raw spectra. Table S3. Results of calibration and prediction PLS1 models for the quantitative compositional analysis of bamboo using pretreated visible-near infrared spectra. Table S4. The experimental values and estimated values for glucose and xylose. [file 13068_2016_443_MOESM1_ESM.docx]

Rapid determination of chemical composition and classification of bamboo fractions using visible-near infrared spectroscopy coupled with multivariate data analysis

Zhong Yang^a,b^, Kang Li^b^, Maomao Zhang ^b^, Donglin Xin^c^, Junhua Zhang^c^

^a^Research Institute of Forestry New Technology, Chinese Academy of Forestry, Beijing 100091, China.

^b^Research Institute of Wood Industry, Chinese Academy of Forestry, Beijing 100091, China.

^c^College of Forestry, Northwest A&F University, 3 Taicheng Road, Yangling 712100, China.

*Corresponding author

junhuazhang@nwsuaf.edu.cn

**Table S1 The experimental values and estimated values for cellulose, xylan and lignin.** A total of samples are 36. M — measured values by wet chemistry measurements, P(raw) — predicted values by raw visible-near infrared spectra, R(raw) — residuals in raw visible-near infrared spectra, P(1st) — predicted values by 1st-derivative pretreated visible-near infrared spectra, R(1st) — residuals in 1st-derivative pretreated visible-near infrared spectra.

| No. of samples | Cellulose | | | | | Xylan | | | | | Lignin | | | | |
| --- | --- | --- | --- | --- | --- | --- | --- | --- | --- | --- | --- | --- | --- | --- | --- |
|  | M | P(raw) | R(raw) | P(1st) | R(1st) | M | P(raw) | R(raw) | P(1st) | R(1st) | M | P(raw) | R(raw) | P(1st) | R(1st) |
| 1 | 49.50 | 48.92 | 0.7 | 49.03 | -0.04 | 24.10 | 25.50 | -1.7 | 26.33 | -1.19 | 16.70 | 18.10 | -2.8 | 16.35 | -1.06 |
| 2 | 49.70 | 50.49 | -1.3 | 51.52 | -0.20 | 30.20 | 28.18 | -0.4 | 29.84 | -0.97 | 13.00 | 15.37 | -1.8 | 13.44 | 0.47 |
| 3 | 51.90 | 49.11 | 0.6 | 51.85 | -0.04 | 30.40 | 30.92 | -1.1 | 26.72 | -0.30 | 15.30 | 15.07 | 2.0 | 20.47 | 1.01 |
| 4 | 55.70 | 51.20 | 5.8 | 53.29 | 2.54 | 30.50 | 29.59 | 0.3 | 32.13 | -1.61 | 11.40 | 12.83 | -1.5 | 12.61 | 0.24 |
| 5 | 52.30 | 48.31 | 4.3 | 52.92 | -0.22 | 25.10 | 23.69 | -0.9 | 22.71 | 0.62 | 20.80 | 18.15 | 4.3 | 20.61 | 0.70 |
| 6 | 46.90 | 51.38 | -4.7 | 47.19 | 0.04 | 43.60 | 40.83 | 1.9 | 44.17 | -0.52 | 3.80 | 4.24 | 0.6 | 3.03 | 0.68 |
| 7 | 44.80 | 51.62 | -7.0 | 46.07 | -0.82 | 43.80 | 40.92 | 2.1 | 42.93 | 0.38 | 5.30 | 4.03 | 2.1 | 4.99 | 0.40 |
| 8 | 49.70 | 50.10 | 0.2 | 50.45 | -0.61 | 41.90 | 41.93 | 0.6 | 41.54 | 0.30 | 4.30 | 2.74 | 1.7 | 4.04 | 0.55 |
| 9 | 48.10 | 52.68 | -2.6 | 47.26 | -0.02 | 35.70 | 41.65 | -4.9 | 36.45 | -0.71 | 5.00 | 3.33 | 1.3 | 3.85 | 1.03 |
| 10 | 52.20 | 49.92 | 1.8 | 52.48 | -0.39 | 41.40 | 41.71 | 0.7 | 40.94 | 0.63 | 1.40 | 1.26 | -1.8 | 2.00 | -1.08 |
| 11 | 52.00 | 50.86 | 0.3 | 51.75 | 0.61 | 43.20 | 39.87 | 1.8 | 43.90 | -0.25 | 2.10 | 4.30 | -1.2 | 1.23 | 0.64 |
| 12 | 54.20 | 49.82 | 4.7 | 52.42 | 1.64 | 42.60 | 41.54 | 2.0 | 42.36 | 0.41 | 1.10 | 1.89 | -1.4 | 2.65 | -1.35 |
| 13 | 56.70 | 48.42 | 8.4 | 58.00 | -1.27 | 37.70 | 40.63 | -1.1 | 36.01 | 1.22 | 1.80 | 1.47 | -1.8 | 3.45 | -2.00 |
| 14 | 48.20 | 49.81 | -1.8 | 47.52 | -0.17 | 22.40 | 24.63 | -1.7 | 22.86 | 0.33 | 18.80 | 19.72 | -1.8 | 19.31 | -0.85 |
| 15 | 47.50 | 48.32 | -1.5 | 46.59 | 0.08 | 26.90 | 26.71 | 1.9 | 28.72 | -0.27 | 17.80 | 17.34 | 0.8 | 18.01 | -1.04 |
| 16 | 50.20 | 49.02 | -0.1 | 50.34 | -0.11 | 26.10 | 27.15 | 0.6 | 26.74 | 0.27 | 16.30 | 16.46 | -1.4 | 14.86 | 0.82 |
| 17 | 49.40 | 49.37 | -0.4 | 50.97 | -1.50 | 27.10 | 26.86 | 2.0 | 25.96 | 0.85 | 15.80 | 15.57 | -2.0 | 14.05 | -0.74 |
| 18 | 45.90 | 50.17 | -3.6 | 45.38 | 0.53 | 22.60 | 22.16 | 0.3 | 21.24 | 0.67 | 21.80 | 20.00 | 3.0 | 23.10 | 0.84 |
| 19 | 35.50 | 35.38 | 0.4 | 32.47 | 0.41 | 19.90 | 20.46 | -3.0 | 21.62 | -0.80 | 26.50 | 25.56 | -0.3 | 28.01 | -0.40 |
| 20 | 35.00 | 31.69 | 6.2 | 36.17 | -0.43 | 20.70 | 18.25 | 3.3 | 19.49 | 0.85 | 21.40 | 20.14 | 0.3 | 22.18 | -0.03 |
| 21 | 38.10 | 31.63 | 5.5 | 38.22 | -0.17 | 19.80 | 20.61 | -2.3 | 23.15 | -1.63 | 26.60 | 21.58 | 7.9 | 24.71 | 1.27 |
| 22 | 33.50 | 32.21 | 1.8 | 34.98 | -0.28 | 19.70 | 18.58 | 1.9 | 19.97 | 0.25 | 21.20 | 20.68 | -2.5 | 18.82 | 0.25 |
| 23 | 34.30 | 35.72 | -0.2 | 35.65 | -0.18 | 21.50 | 17.78 | 2.8 | 20.40 | 1.21 | 23.70 | 26.62 | -0.4 | 25.32 | -0.17 |
| 24 | 27.50 | 30.76 | -2.1 | 27.76 | -0.25 | 24.80 | 26.89 | -0.4 | 25.40 | -0.59 | 5.20 | 5.40 | -1.0 | 5.69 | -0.34 |
| 25 | 23.60 | 29.56 | -6.6 | 24.80 | -1.12 | 26.30 | 24.87 | 0.6 | 25.29 | 0.38 | 9.80 | 10.47 | 0.1 | 9.61 | -0.11 |
| 26 | 29.60 | 29.41 | -0.5 | 28.60 | 0.71 | 25.20 | 24.88 | -0.9 | 24.66 | 0.32 | 6.80 | 7.03 | 0.9 | 6.45 | 0.43 |
| 27 | 34.80 | 28.90 | 4.4 | 34.49 | 0.53 | 25.90 | 24.11 | 0.4 | 25.19 | 0.64 | 11.00 | 7.76 | 1.6 | 8.79 | 2.12 |
| 28 | 22.80 | 26.14 | -3.5 | 23.83 | -0.47 | 21.30 | 24.44 | -2.4 | 22.70 | -0.65 | 1.80 | 0.30 | 0.6 | 1.36 | 1.04 |
| 29 | 27.00 | 27.41 | 0.1 | 25.30 | 1.05 | 28.20 | 25.23 | 4.7 | 25.35 | 2.03 | 4.10 | 8.06 | -4.7 | 6.18 | -2.50 |
| 30 | 26.70 | 28.26 | -2.2 | 26.47 | 0.13 | 24.00 | 25.20 | -2.4 | 25.06 | -0.81 | 2.50 | 3.85 | 0.1 | 2.52 | -0.21 |
| 31 | 31.30 | 26.95 | 2.1 | 31.27 | 0.14 | 25.20 | 23.65 | -1.5 | 26.34 | -0.60 | 3.90 | 5.49 | -0.9 | 4.10 | -0.03 |
| 32 | 35.00 | 35.49 | 0.7 | 35.74 | -0.43 | 20.50 | 21.24 | 2.2 | 21.83 | -0.82 | 26.30 | 25.07 | -0.3 | 24.85 | 0.00 |
| 33 | 32.30 | 35.81 | -3.7 | 33.25 | -0.47 | 17.50 | 20.32 | -2.2 | 17.89 | -0.25 | 21.40 | 21.90 | 0.6 | 20.45 | 1.56 |
| 34 | 33.50 | 33.91 | -0.8 | 34.15 | -0.91 | 17.20 | 20.25 | -3.0 | 17.59 | -0.56 | 22.30 | 22.03 | 1.3 | 22.84 | -0.59 |
| 35 | 32.70 | 36.35 | -4.3 | 30.52 | 1.38 | 18.10 | 19.54 | -1.5 | 16.69 | 0.61 | 22.60 | 23.37 | 0.7 | 23.85 | -0.67 |
| 36 | 33.30 | 36.10 | -1.2 | 32.79 | 0.29 | 17.30 | 17.64 | 1.4 | 15.66 | 0.55 | 26.30 | 28.74 | -2.1 | 27.43 | -0.89 |

**Table S2 Results of calibration and prediction PLS2 models for the quantitative compositional analysis of bamboo using raw spectra.** C+X — cellulose and xylan were modeled simultaneously, C+L — cellulose and lignin were modeled simultaneously, L+C — lignin and cellulose were modeled simultaneously, C+X+L — cellulose, xylan and lignin were modeled simultaneously, G+X — glucose and xylose were modeled simultaneously.

| Wavelength(nm) | Models | Chemical composition | R^2^c | RMSEC | R^2^p | RMSEP | SD | RPD | RER |
| --- | --- | --- | --- | --- | --- | --- | --- | --- | --- |
| 400-780 | Model 1 (C+X) | Cellulose | 0.84 | 4.0 | 0.75 | 5.1 | 9.4 | 1.8 | 5.4 |
|  |  | Xylan | 0.96 | 1.7 | 0.90 | 2.6 | 8.5 | 3.3 | 11.1 |
|  | Model 2 (C+L) | Cellulose | 0.77 | 4.9 | 0.66 | 6.0 | 9.4 | 1.6 | 5.3 |
|  |  | Lignin | 0.86 | 3.2 | 0.77 | 4.2 | 8.6 | 2.0 | 7.2 |
|  | Model 3 (L+C) | Xylan | 0.88 | 2.8 | 0.83 | 3.4 | 8.0 | 2.3 | 8.4 |
|  |  | Lignin | 0.85 | 3.1 | 0.77 | 4.1 | 8.3 | 2.0 | 6.9 |
|  | Model 4 (C+X+L) | Cellulose | 0.78 | 4.8 | 0.63 | 6.4 | 14.6 | 2.3 | 5.0 |
|  |  | Xylan | 0.91 | 2.5 | 0.85 | 3.3 | 8.0 | 2.5 | 8.6 |
|  |  | Lignin | 0.86 | 3.2 | 0.77 | 4.3 | 8.3 | 2.0 | 7.0 |
|  | Model 5 (G+X) | Glucose | 0.91 | 5.5 | 0.72 | 9.8 | 17.4 | 1.8 | 6.0 |
|  |  | Xylose | 0.96 | 4.3 | 0.87 | 8.6 | 23.6 | 2.8 | 9.1 |
| 780-2500 | Model 1 (C+X) | Cellulose | 0.97 | 1.7 | 0.94 | 2.5 | 10.1 | 4.1 | 13.3 |
|  |  | Xylan | 0.97 | 1.4 | 0.91 | 2.5 | 8.6 | 3.4 | 10.8 |
|  | Model 2 (C+L) | Cellulose | 0.96 | 2.0 | 0.92 | 2.8 | 10.0 | 3.6 | 10.1 |
|  |  | Lignin | 0.96 | 1.6 | 0.92 | 2.6 | 8.8 | 3.4 | 11.0 |
|  | Model 3 (L+C) | Xylan | 0.92 | 2..356 | 0.87 | 3.0 | 8.2 | 2.7 | 9.5 |
|  |  | Lignin | 0.94 | 2.1 | 0.91 | 2.6 | 8.6 | 3.4 | 10.6 |
|  | Model 4 (C+X+L) | Cellulose | 0.88 | 3.6 | 0.81 | 4.4 | 14.6 | 3.3 | 6.3 |
|  |  | Xylan | 0.92 | 2.4 | 0.86 | 3.1 | 8.0 | 2.6 | 9.3 |
|  |  | Lignin | 0.94 | 2.1 | 0.90 | 2.7 | 8.6 | 3.2 | 9.7 |
|  | Model 5 (G+X) | Glucose | 0.84 | 7.0 | 0.72 | 9.5 | 16.8 | 1.8 | 5.9 |
|  |  | Xylose | 0.90 | 7.3 | 0.79 | 10.9 | 23.4 | 2.2 | 7.0 |
| 400-2500 | Model 1 (C+X) | Cellulose | 0.87 | 3.6 | 0.83 | 4.2 | 9.6 | 2.3 | 6.0 |
|  |  | Xylan | 0.94 | 2.1 | 0.90 | 2.6 | 8.2 | 3.2 | 9.9 |
|  | Model 2 (C+L) | Cellulose | 0.88 | 3.4 | 0.83 | 4.2 | 9.7 | 2.3 | 6.5 |
|  |  | Lignin | 0.91 | 2.6 | 0.87 | 3.1 | 8.5 | 2.7 | 9.6 |
|  | Model 3 (L+C) | Xylan | 0.94 | 2.1 | 0.89 | 2.7 | 8.2 | 3.1 | 9.6 |
|  |  | Lignin | 0.91 | 2.6 | 0.87 | 3.1 | 8.6 | 2.7 | 9.8 |
|  | Model 4 (C+X+L) | Cellulose | 0.88 | 3.6 | 0.82 | 4.3 | 14.6 | 3.4 | 6.0 |
|  |  | Xylan | 0.93 | 2.1 | 0.90 | 2.6 | 8.1 | 3.2 | 9.8 |
|  |  | Lignin | 0.88 | 3.0 | 0.81 | 3.8 | 8.3 | 2.2 | 7.3 |
|  | Model 5 (G+X) | Glucose | 0.84 | 7.2 | 0.73 | 9.3 | 16.8 | 1.8 | 6.0 |
|  |  | Xylose | 0.87 | 8.4 | 0.75 | 11.8 | 22.5 | 1.9 | 5.9 |

**Table S3 Results of calibration and prediction PLS1 models for the quantitative compositional analysis of bamboo using pretreated visible-near infrared spectra.** C+X — cellulose and xylan were modeled simultaneously, C+L — cellulose and lignin were modeled simultaneously, L+C — lignin and cellulose were modeled simultaneously, C+X+L — cellulose, xylan and lignin were modeled simultaneously, G+X — glucose and xylose were modeled simultaneously.

| Pretreatment | Models | Chemical composition | Factors | R^2^_C_ | RMSEC | R^2^_P_ | RMSEP | SD | RPD | RER |
| --- | --- | --- | --- | --- | --- | --- | --- | --- | --- | --- |
| MSC | Model 1 (C+X) | Cellulose | 5 | 0.897 | 3.229 | 0.856 | 3.8 | 9.8 | 2.5 | 7.1 |
|  |  | Xylan | 5 | 0.941 | 1.91 | 0.916 | 2.4 | 8.2 | 3.4 | 11.2 |
|  | Model 2 (C+L) | Cellulose | 5 | 0.895 | 3.256 | 0.854 | 3.9 | 9.8 | 2.5 | 7.2 |
|  |  | Lignin | 5 | 0.903 | 2.69 | 0.865 | 3.2 | 8.5 | 2.6 | 9.2 |
|  | Model 3 (L+C) | Xylan | 4 | 0.939 | 2.01 | 0.912 | 2.5 | 8.2 | 3.3 | 10.9 |
|  |  | Lignin | 4 | 0.895 | 2.82 | 0.861 | 3.3 | 8.6 | 2.6 | 9.2 |
|  | Model 4 (C+X+L) | Cellulose | 5 | 0.891 | 3.3 | 0.848 | 3.9 | 9.7 | 2.5 | 7.3 |
|  |  | Xylan | 5 | 0.945 | 1.9 | 0.914 | 2.4 | 8.1 | 3.3 | 10.9 |
|  |  | Lignin | 5 | 0.903 | 2.7 | 0.861 | 3.3 | 8.5 | 2.6 | 9.2 |
|  | Model 5 (G+X) | Glucose | 17 | 0.996 | 0.8 | 0.968 | 3.3 | 14.5 | 4.4 | 19.4 |
|  |  | Xylose | 17 | 0.998 | 0.7 | 0.982 | 3.1 | 20.9 | 6.6 | 22.8 |
| EMSC | Model 1 (C+X) | Cellulose | 4 | 0.901 | 3.2 | 0.865 | 3.7 | 9.8 | 2.6 | 8.3 |
|  |  | Xylan | 4 | 0.947 | 1.9 | 0.924 | 2.3 | 8.2 | 3.6 | 12.3 |
|  | Model 2 (C+L) | Cellulose | 11 | 0.955 | 2.1 | 0.916 | 3.0 | 10.2 | 3.4 | 11.6 |
|  |  | Lignin | 11 | 0.976 | 1.3 | 0.929 | 2.3 | 8.8 | 3.8 | 12.0 |
|  | Model 3 (L+C) | Xylan | 8 | 0.966 | 1.5 | 0.929 | 2.2 | 8.3 | 3.8 | 12.0 |
|  |  | Lignin | 8 | 0.966 | 1.6 | 0.931 | 2.3 | 8.8 | 3.9 | 12.7 |
|  | Model 4 (C+X+L) | Cellulose | 3 | 0.857 | 3.8 | 0.819 | 4.3 | 9.5 | 2.2 | 6.8 |
|  |  | Xylan | 3 | 0.943 | 2.0 | 0.924 | 2.3 | 8.1 | 3.6 | 12.3 |
|  |  | Lignin | 3 | 0.878 | 3.1 | 0.850 | 3.4 | 8.3 | 2.4 | 8.1 |
|  | Model 5 (G+X) | Glucose | 18 | 0.998 | 0.3 | 0.994 | 1.4 | 18.7 | 13.1 | 46.7 |
|  |  | Xylose | 18 | 0.998 | 0.3 | 0.992 | 2.1 | 24.1 | 11.3 | 34.3 |
| SNV | Model 1 (C+X) | Cellulose | 5 | 0.899 | 3.2 | 0.859 | 3.8 | 9.8 | 2.6 | 7.3 |
|  |  | Xylan | 5 | 0.941 | 2.0 | 0.910 | 2.5 | 8.2 | 3.3 | 10.6 |
|  | Model 2 (C+L) | Cellulose | 5 | 0.901 | 3.2 | 0.861 | 3.8 | 9.8 | 2.6 | 7.2 |
|  |  | Lignin | 5 | 0.908 | 2.5 | 0.870 | 3.2 | 8.6 | 2.7 | 9.4 |
|  | Model 3 (L+C) | Xylan | 6 | 0.947 | 1.9 | 0.908 | 2.5 | 8.3 | 3.3 | 11.0 |
|  |  | Lignin | 6 | 0.931 | 2.3 | 0.903 | 2.7 | 8.7 | 3.2 | 10.2 |
|  | Model 4 (C+X+L) | Cellulose | 5 | 0.895 | 3.3 | 0.854 | 3.9 | 9.7 | 2.5 | 7.1 |
|  |  | Xylan | 5 | 0.941 | 2.0 | 0.908 | 2.5 | 8.1 | 3.2 | 10.4 |
|  |  | Lignin | 5 | 0.904 | 2.7 | 0.865 | 3.2 | 8.5 | 2.6 | 9.2 |
|  | Model 5 (G+X) | Glucose | 19 | 0.998 | 0.4 | 0.978 | 2.8 | 19.1 | 6.9 | 25.2 |
|  |  | Xylose | 19 | 0.998 | 0.2 | 0.996 | 1.2 | 24.0 | 19.3 | 51.2 |
| first derivative | Model 1 (C+X) | Cellulose | 10 | 0.994 | 0.7 | 0.988 | 1.1 | 10.3 | 9.3 | 31.6 |
|  |  | Xylan | 10 | 0.988 | 0.9 | 0.974 | 1.3 | 8.3 | 6.4 | 21.5 |
|  | Model 2 (C+L) | Cellulose | 11 | 0.994 | 0.7 | 0.891 | 1.1 | 10.2 | 9.4 | 31.4 |
|  |  | Lignin | 11 | 0.994 | 0.7 | 0.976 | 1.3 | 9.0 | 6.9 | 20.3 |
|  | Model 3 (L+C) | Xylan | 8 | 0.980 | 1.1 | 0.960 | 1.6 | 8.4 | 5.1 | 15.6 |
|  |  | Lignin | 8 | 0.982 | 1.1 | 0.970 | 1.5 | 8.9 | 6.0 | 18.1 |
|  | Model 4 (C+X+L) | Cellulose | 12 | 0.996 | 0.7 | 0.986 | 1.2 | 10.2 | 8.7 | 28.5 |
|  |  | Xylan | 12 | 0.990 | 0.8 | 0.968 | 1.5 | 8.3 | 5.7 | 19.0 |
|  |  | Lignin | 12 | 0.994 | 0.7 | 0.974 | 1.4 | 8.9 | 6.2 | 17.9 |
|  | Model 5 (G+X) | Glucose | 8 | 0.992 | 1.6 | 0.976 | 2.8 | 17.9 | 6.4 | 20.3 |
|  |  | Xylose | 8 | 0.996 | 1.5 | 0.988 | 2.5 | 23.5 | 9.4 | 26.9 |
| second derivative | Model 1 (C+X) | Cellulose | 10 | 0.990 | 1.0 | 0.976 | 1.5 | 10.2 | 6.8 | 19.9 |
|  |  | Xylan | 10 | 0.988 | 0.9 | 0.970 | 1.4 | 8.4 | 6.0 | 20.7 |
|  | Model 2 (C+L) | Cellulose | 12 | 0.990 | 0.9 | 0.972 | 1.7 | 10.4 | 6.1 | 19.0 |
|  |  | Lignin | 12 | 0.994 | 0.6 | 0.982 | 1.2 | 9.0 | 7.7 | 20.9 |
|  | Model 3 (L+C) | Xylan | 12 | 0.992 | 0.7 | 0.982 | 1.1 | 8.4 | 7.6 | 26.7 |
|  |  | Lignin | 12 | 0.994 | 0.6 | 0.988 | 0.9 | 8.9 | 10.0 | 28.6 |
|  | Model 4 (C+X+L) | Cellulose | 13 | 0.992 | 0.9 | 0.972 | 1.7 | 10.2 | 5.8 | 17.9 |
|  |  | Xylan | 13 | 0.988 | 0.9 | 0.955 | 1.8 | 8.3 | 4.7 | 16.2 |
|  |  | Lignin | 13 | 0.992 | 0.8 | 0.970 | 1.6 | 8.9 | 5.7 | 15.5 |
|  | Model 5 (G+X) | Glucose | 7 | 0.988 | 1.9 | 0.978 | 2.6 | 18.0 | 6.8 | 23.2 |
|  |  | Xylose | 7 | 0.992 | 2.1 | 0.984 | 3.0 | 23.6 | 7.9 | 23.0 |

**Table S4 The experimental values and estimated values for glucose and xylose.** A total of samples are 26. M — measured values by wet chemistry measurements, P(raw) — predicted values by raw visible-near infrared spectra, R(raw) — residuals in raw visible-near infrared spectra, P(1st) — predicted values by first derivative pretreated visible-near infrared spectra, R(1st) — residuals in first derivative pretreated visible-near infrared spectra.

| No. of samples | Glucose | | | | | Xylose | | | | |
| --- | --- | --- | --- | --- | --- | --- | --- | --- | --- | --- |
|  | M | P(raw) | R(raw) | P(1st) | R(1st) | M | P(raw) | R(raw) | P(1st) | R(1st) |
| 1 | 51.56 | 60.10 | -8.5 | 48.52 | -0.67 | 57.06 | 66.55 | -8.3 | 55.82 | 1.09 |
| 2 | 90.30 | 79.00 | 11.3 | 90.70 | 0.57 | 88.23 | 86.24 | 8.5 | 87.00 | 0.46 |
| 3 | 67.15 | 75.57 | -8.4 | 73.90 | 0.01 | 69.94 | 84.70 | -9.5 | 69.35 | 0.08 |
| 4 | 77.21 | 79.88 | -2.7 | 77.44 | 0.15 | 76.61 | 83.90 | -3.3 | 79.02 | -0.15 |
| 5 | 44.85 | 54.85 | -10.0 | 45.68 | -0.55 | 51.89 | 61.10 | -4.6 | 48.89 | 0.93 |
| 6 | 96.11 | 101.29 | -5.2 | 96.51 | -0.10 | 96.40 | 102.28 | -3.9 | 95.06 | 0.21 |
| 7 | 100.53 | 98.90 | 1.6 | 100.76 | -0.78 | 101.60 | 100.82 | 3.3 | 101.11 | 0.33 |
| 8 | 100.07 | 104.96 | -4.9 | 101.23 | -0.37 | 101.12 | 102.09 | -1.2 | 99.30 | 0.16 |
| 9 | 102.84 | 95.68 | 7.2 | 102.43 | 0.09 | 102.73 | 94.33 | 9.2 | 105.58 | -0.22 |
| 10 | 86.91 | 86.81 | 0.1 | 87.92 | -0.01 | 89.57 | 91.58 | -8.0 | 89.60 | -0.72 |
| 11 | 99.62 | 97.37 | 2.2 | 98.61 | 0.61 | 98.92 | 99.61 | 1.9 | 100.40 | -0.42 |
| 12 | 99.19 | 98.46 | 0.7 | 99.72 | 0.27 | 99.20 | 97.68 | -1.5 | 97.96 | 0.22 |
| 13 | 100.22 | 99.53 | 0.7 | 98.50 | 0.02 | 100.08 | 94.81 | 2.7 | 101.11 | -0.03 |
| 14 | 52.21 | 49.92 | 2.3 | 52.61 | 0.74 | 71.30 | 62.45 | 7.0 | 71.05 | -1.32 |
| 15 | 67.24 | 66.75 | 0.5 | 63.92 | -0.72 | 84.24 | 77.11 | -0.6 | 80.98 | 1.03 |
| 16 | 75.84 | 67.74 | 8.1 | 76.47 | 0.21 | 92.79 | 79.18 | 9.8 | 95.27 | -0.53 |
| 17 | 76.17 | 69.01 | 7.2 | 77.57 | 0.12 | 91.85 | 78.76 | 7.8 | 91.47 | -0.29 |
| 18 | 45.15 | 48.11 | -3.0 | 48.57 | 0.09 | 51.92 | 61.10 | -8.0 | 56.62 | -0.59 |
| 19 | 71.28 | 81.74 | -10.5 | 67.15 | 0.33 | 43.78 | 54.61 | -12.5 | 41.64 | 2.58 |
| 20 | 90.37 | 84.14 | 6.2 | 85.19 | 0.92 | 60.56 | 56.84 | 4.5 | 57.70 | 2.72 |
| 21 | 66.20 | 78.99 | -12.8 | 65.43 | 0.72 | 39.19 | 53.07 | -12.7 | 40.94 | -0.78 |
| 22 | 64.72 | 66.90 | -2.2 | 67.56 | -0.72 | 38.94 | 42.15 | -10.6 | 45.51 | -1.63 |
| 23 | 72.14 | 61.42 | 10.7 | 70.58 | 0.46 | 37.74 | 34.00 | 6.7 | 38.36 | -0.03 |
| 24 | 85.95 | 77.43 | 8.5 | 91.04 | -2.09 | 58.95 | 47.27 | 9.5 | 59.47 | -1.36 |
| 25 | 61.96 | 71.06 | -9.1 | 67.95 | -0.24 | 39.73 | 44.77 | 1.3 | 43.31 | -2.72 |
| 26 | 71.95 | 62.13 | 9.8 | 72.40 | 0.95 | 49.52 | 36.86 | 12.3 | 45.66 | 0.97 |
